# Human Genetic Adaptation to High Altitude: Evidence from the Andes

**DOI:** 10.3390/genes10020150

**Published:** 2019-02-15

**Authors:** Colleen G. Julian, Lorna G. Moore

**Affiliations:** 1 Department of Medicine, University of Colorado Anschutz Medical Campus, Aurora, CO 80045, USA; 2 Department of Obstetrics and Gynecology, University of Colorado Anschutz Medical Campus, Aurora, CO 80045, USA; lorna.Moore@ucdenver.edu

**Keywords:** adaptation, hypoxia, altitude, genomics, epigenomics

## Abstract

Whether Andean populations are genetically adapted to high altitudes has long been of interest. Initial studies focused on physiological changes in the O_2_ transport system that occur with acclimatization in newcomers and their comparison with those of long-resident Andeans. These as well as more recent studies indicate that Andeans have somewhat larger lung volumes, narrower alveolar to arterial O_2_ gradients, slightly less hypoxic pulmonary vasoconstrictor response, greater uterine artery blood flow during pregnancy, and increased cardiac O_2_ utilization, which overall suggests greater efficiency of O_2_ transfer and utilization. More recent single nucleotide polymorphism and whole-genome sequencing studies indicate that multiple gene regions have undergone recent positive selection in Andeans. These include genes involved in the regulation of vascular control, metabolic hemostasis, and erythropoiesis. However, fundamental questions remain regarding the functional links between these adaptive genomic signals and the unique physiological attributes of highland Andeans. Well-designed physiological and genome association studies are needed to address such questions. It will be especially important to incorporate the role of epigenetic processes (i.e., non-sequence-based features of the genome) that are vital for transcriptional responses to hypoxia and are potentially heritable across generations. In short, further exploration of the interaction among genetic, epigenetic, and environmental factors in shaping patterns of adaptation to high altitude promises to improve the understanding of the mechanisms underlying human adaptive potential and clarify its implications for human health.

## 1. Introduction

Since the early 1900s, anthropologists and physiologists alike have sought to determine if there has been genetic adaptation to high altitude, conventionally defined as above 2500 m or 8250 ft since that is where O_2_ saturation levels in the arterial blood begin to fall in most persons. Some of the first studies took place in the Andes, where approximately 6 million indigenous Aymara and Quechua (whom we shall refer to here as Andeans) populations reside, chiefly in Peru and Bolivia, however also in neighboring countries. Andeans are derived from the early settlers of the Americas who reached South America 15 to 16 thousand years ago (kya) and then split into two branches, one that settled in the Pacific coastal and Andean regions and the other that moved along the Atlantic coast and then eastward [1]. Of interest, there has been little admixture between Andeans with the descendants of the more easterly groups as attested to by mitochondrial and autosomal genetic markers, demonstrating the Andeans’ genetic continuity and substantial isolation from other South American groups [1,2].

Initial debate as to whether genetic adaptation to high altitude has taken place was driven by differences in theoretical orientation and the sources of evidence being considered. Theoretical orientation influenced the way in which the term “adaptation” was being employed. Physiologists used the term to refer to any response regardless of whether it was likely to be beneficial or otherwise affect the chance(s) of being able to live or reproduce, whereas evolutionary biologists or geneticists restricted its usage to those responses likely to influence reproductive success [3]. Evidence for adaptation was predominantly sought from studies separating short-term physiological responses or those occurring over hours to days or even weeks, termed acclimatization, from those occurring across lifetimes, termed developmental responses, and from those that persisted independent of duration of high-altitude exposure and were inferred to be genetic. The migration model, introduced in the 1960s, was productively used to distinguish between acclimatization, developmental, and presumed genetic responses [4,5,6]. The advent of single nucleotide polymorphism technologies and statistical methods for detecting evidence of natural selection constituted a paradigm shift and resulted in an exponential rise in the number of publications reporting genetic adaptation [7]. While multiple studies have shown that Andean and other high-altitude populations have undergone natural selection in several gene regions influencing O_2_-sensitive pathways, numerous questions remain regarding the biological processes driving human adaptation to the chronic hypoxia of high altitude and their importance for human health.

This review discusses the kinds of evidence by which adaptation to high altitude has been assessed and which have led to widespread acceptance of the idea that genetic adaptation to high altitude has occurred. Studies in Andean residents of high altitude are summarized with respect to the physiological characteristics distinguishing them from acclimatized newcomers and the genomic or genetic factors that are potentially involved. While further study is needed, such studies offer the opportunity to identify the importance of interactions between genomic and epigenomic processes for human adaptation to limited oxygen availability.

## 2. Genetic Adaptation of Andean High-Altitude Populations

Two kinds of information support the existence of Andean genetic adaptation to high altitude. First, indirect evidence provided by physiologic studies demonstrates that native highland populations exhibit unique O_2_ transport traits when compared with acclimatized newcomers that cannot be attributed to developmental processes. Second, direct evidence comes from genomic studies that show signals of recent positive selection in specific gene regions. However, despite the remarkable progress in recent years for identifying targets of natural selection and the recognition that many are involved in O_2_-sensitive signaling pathways, few investigations have been able to show how these gene regions affect specific physiological characteristics and how, in turn, such effects influence reproductive success. From an evolutionary point of view, these relationships are essential since, by definition, only genes with effects on reproductive success are acted upon by natural selection.

### 2.1. Physiologic Evidence of Genetic Adaptation to High Altitude

Since the hypoxia of high altitude challenges O_2_ homeostasis, there has been a long history of studies of the O_2_ transport system and its components (arterial O_2_ content, distribution, and utilization) in acclimatized newcomers, Andeans, and other long-term residents of high altitude for establishing the physiologic mechanisms underlying human adaptation to high altitude.

#### 2.1.1. O_2_ Content

**Figure 1:**
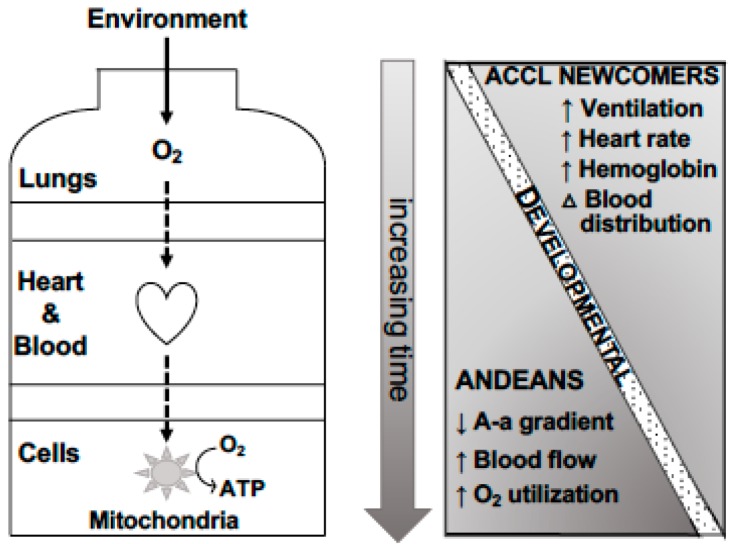
The O_2_ transport system and its temporal changes. The O_2_ transport system consists of two pumps (the lungs and the heart), two diffusion steps (alveoli to arterial blood, capillary blood to the mitochondria), and the mitochondria where O_2_ is consumed to generate chemical energy, adenosine triphosphate (ATP). Increases in the levels of ventilation, heart rate, and hemoglobin as well as changes in blood flow distribution to favor high demand organs occur with acclimatization. Developmental changes increase lung volume. In Andeans, multigenerational high-altitude residence produces further changes in the alveoli to arterial O_2_ gradient, regional blood flow, and O_2_ utilization. See text for details and references. Adapted from [12].

The partial pressure of O_2_ in the arterial blood (P_a_O_2_) is determined by alveolar ventilation and the alveolar-arterial (A-a) O_2_ gradient (Figure 1). Since the A-a O_2_ gradient is minimal in healthy persons, alveolar or end-tidal PCO_2_ (P_A_CO_2_) can serve as a proxy for arterial PCO_2_ (P_a_CO_2_). Additionally, since according to the alveolar air equation P_a_CO_2_ is inversely related to alveolar ventilation, P_A_CO_2_ can serve as an index of alveolar ventilation per unit of CO_2_ production (or metabolic rate given that, normally, CO_2_ production and O_2_ consumption are closely coupled). At low altitude, P_A_CO_2_ averages ~40 mmHg, however it falls to ~10 mmHg at elevations above 3000 m and even further at extreme altitudes. P_A_CO_2_ or alveolar ventilation is lower in Andeans and acclimatized newcomers than sea-level residents, however Andean values are somewhat higher than those of acclimatized newcomers, indicating lower levels of alveolar ventilation (Table 1) [8,9,10]. Consistent with this, the hypoxic ventilatory response of Andeans is blunted compared to acclimatized newcomers. Indicating a genetic component, greater indigenous (Quechua) ancestry is directly related to the blunting that is observed [11].

Acclimatization does not appreciably change the A-a O_2_ gradient, however the gradient is lower in Andeans (and other lifelong of high- compared to low-altitude residents), enabling Andeans to achieve an increased efficiency of O_2_ transfer [13,14,15]. This enhanced efficiency is likely the result of greater total lung capacity and, especially, residual volume in Andean populations compared with those residing at low altitudes [16] and gives rise to the “barrel-chest” Andean morphology. Developmental processes play a key role in such lung-volume expansion with increased lung growth being apparent from infancy through adolescence not only in Andeans, however also other high-altitude residents, including Coloradans and even experimental animals born and raised under hypoxic conditions [17,18]. Genetic factors are also implicated insofar as Andean total lung volumes are greater than values seen in lowlanders born and raised at high altitude [19], and the proportion of indigenous American ancestry is directly related to residual volume yet, interestingly, not chest dimensions [20]. However, there appears to be an interaction between Andean ancestry and high-altitude residence that acts to increase the altitude-associated expansion of chest dimensions and lung volumes [19] and reduction in stature and limb measurements [21]. While more studies are needed with controls for confounding factors, the improved efficiency of O_2_ transfer is likely important for maintaining arterial O_2_ saturation and thus, blood O_2_ content during exercise [16,22].

The position of the hemoglobin-O_2_ dissociation curve determines the level of arterial O_2_ saturation (SaO_2_) for a given PaO_2_. Little change occurs in the in vivo curve position with acclimatization since the left curve-shifting effect of respiratory alkalosis is offset by an increase in red blood cell 2,3-bisphosphoglyceric acid levels. Current evidence does not support differences in Andean versus acclimatized newcomer curve position (J. Prchal, personal communication) [23,24,25] (Table 1). Of interest, the I-allele of the angiotensin-converting enzyme has been associated with higher SaO_2_ in Quechua regardless of whether they were born at high altitude or at low altitude yet were exposed transiently to high altitude [26], however this allele is not unique to highlanders as it is found in human populations worldwide.

Hemoglobin levels rise with ascent to high altitude in both men and women, due initially to a contraction in plasma volume and subsequently to a rise in red blood cell production. Generally, hemoglobin levels in acclimatized newcomers are similar to those present in healthy Andeans (Table 1). Urban values are higher than those of rural residents [27,28], perhaps due to dust exposure [29] or the greater levels of admixture present in Andean mining communities [30]. While modest increases in hemoglobin and red cell production are considered beneficial at high altitude, excessive erythrocytosis is maladaptive; a detailed discussion of this topic is provided in Section 2.2.2.

Arterial O_2_ content (CaO_2_) is either measured directly or calculated using hemoglobin values (in gm/dL multiplied by 1.36, the mls of O_2_ bound per gm) multiplied by SaO_2_, using a correction factor for dissolved O_2_. Acclimatized newcomers re-establish the sea-level value of ~21 vol % due to their hyperventilation, which helps to overcome the initial fall in PaO_2_, and increased hemoglobin. Andean and acclimatized newcomers achieve similar CaO_2_ levels due to the Andeans’ narrower alveolar-arterial O_2_ gradient offsetting their somewhat lower levels of ventilation.

#### 2.1.2. O_2_ Distribution

Acclimatized newcomers and Andeans have similar levels of cardiac output at a given workload, however values are lower at maximal exercise in both groups compared to sea level [31]. Despite considerable research, the cause of the reduction in maximal cardiac output remains unclear, leading some to suggest that cardiac output is actively suppressed by central nervous system factors [31,32]. Decreased filling is unlikely since low- and high-altitude residents do not differ in terms of blood volume [33]. Increased afterload due to higher pulmonary arterial pressures could be a factor, however values are lower in healthy Andeans compared with acclimatized newcomers and systemic (left heart) pressures are modestly lower in Andeans yet cardiac output is similar [34,35]. While continuing to be debated, a substantial number of studies indicate that Andeans have higher maximal O_2_ consumption than acclimatized newcomers and less altitude-associated decrement [36].

O_2_ distribution is determined by regional blood flow. There is increased sympathetic nervous system stimulation in acclimatized lowlanders at high altitude which likely reduced blood flow to the periphery [44,45]. Blood flow to the leg and fractional O_2_ extraction during exercise are reduced in Andeans compared with acclimatized lowlanders as a result of blood being diverted to other tissues [46]. Blood flow velocity through the internal carotid, middle cerebral, and vertebral arteries has been used as indices of brain blood flow; however it should be recognized that blood flow is also a function of the vessel diameter or cross-sectional area. Highland Andeans appear to have lower resting middle cerebral flow velocities than at low altitude, however unchanged O_2_ delivery due to higher hemoglobin levels [39,47] (Table 1); the adaptive significance of reduced middle cerebral blood flow, however, is not clear. Cerebral blood vessels are highly responsive to blood gas changes or bioactive molecules such as NO. Andeans had less middle cerebral artery vasodilator response to hypoxia or NO at high compared to low altitude, and less vasoconstrictor response to hypocapnia than Sherpa [48]; however whether such responses differed from those of acclimatized newcomers was not studied. Compared to acclimatized lowlanders, Andeans distribute a larger proportion of pelvic blood flow to the uteroplacental circulation during pregnancy, which in turn raises uterine artery blood flow and uteroplacental O_2_ delivery [49,50,51] (Table 1). Andean protection was accompanied by greater antioxidant levels and more angiogenic relative to anti-angiogenic substances [52,53]. Developmental factors were not responsible since the Andeans’ uterine artery blood flow was greater than that seen in Europeans who were born and raised at high altitude [54]. Cortisol levels are also lower in pregnant Andeans than acclimatized newcomers [55], perhaps reflecting less sympathetic stimulation. Greater vascularity, as observed in the placental [56] and the skin microcirculation in neonates [57], both could also increase blood flow.

#### 2.1.3. O_2_ Utilization

O_2_ delivery to the mitochondria generates chemical energy or ATP (adenosine triphosphate) (Figure 1). Of interest is that the efficiency with which ATP is produced varies by fuel source. Specifically, the metabolism of carbohydrates (glucose, glycogen) generates 25–50% more ATP per mole of O_2_ consumed than is the case with the use of free fatty acids or lipids [58]. Carbohydrates become the preferred fuel in males after three weeks of altitude acclimatization [59]; however, interestingly, not for females [60]. Only a few studies have been performed in long-term high-altitude residents. Specifically, using positron emission tomography to measure heart metabolism, Hochachka and co-workers found greater reliance on carbohydrate metabolism in Quechua males studied at sea level and 50–60% more ATP produced per mole of O_2_ consumption compared with lowlanders [58]. The authors concluded that Quechua hearts displayed increased O_2_ efficiency, representing a biochemical adaptation for defending against hypoxia [32]. Residence at high altitude also alters glucose metabolism. Glucose uptake is increased, glucose tolerance is improved, and consequently, venous glucose levels are lower at high altitude [61,62] as well as in pregnant Andeans [63]; this was interpreted as reflecting greater placenta glucose uptake in order to spare O_2_ for fetal consumption [64].

In summary, the several unique O_2_ transport characteristics of Andeans compared to acclimatized newcomers that are not due to developmental factors—namely, lower alveolar ventilation, lower pulmonary vasoconstrictor response, larger lung volumes, higher uterine artery and possibly lower middle cerebral blood flow, less altitude decrement in maximal exercise O_2_ consumption, and more efficient cardiac O_2_ utilization—suggest a greater efficiency of O_2_ transfer and utilization and are consistent with the likelihood of Andean genetic adaptation to high altitude.

### 2.2. Genomic Evidence of Andean High Altitude Adaptation

Direct evidence for Andean genetic adaptation to high altitude comes from single nucleotide polymorphism (SNP) genome scans and sequencing studies that have identified genomic regions with evidence of recent positive selection (Table 2). Genes that regulate or are regulated by the hypoxia-inducible factor (HIF) pathway have been of particular interest. HIF consists of two α-subunits (HIF1α and HIF2α) and a constitutively expressed β-subunit [65,66]. In normoxia, O_2_-dependent negative regulators of HIF called prolyl hydroxylases (PHDs) enable the hydroxylation of proline residues of HIF1/2α subunits [67]; this promotes the binding of von Hippel–Lindau tumor suppressor (vHL) protein and, subsequently, degrades the HIF1/2α [68,69]. In a hypoxic environment, HIF1/2α are not hydroxylated by PH and therefore escape recognition by vHL, allowing these subunits to bind with hypoxia responsive elements (HRE) within gene promoters and associated cofactors to initiate HIF-regulated gene transcription [70]. While there are more than 100 genes containing response elements to which HIF can bind, existing SNP data indicate that the HIF-pathway has not been disproportionately acted upon by natural selection [71]. Further, not all O_2_-sensitive genes contain HREs. Therefore HIF, while certainly central for governing transcriptional responses to hypoxia, is not the *only* regulator of molecular responses to changes in O_2_ tension.

The first genome scan to study high-altitude adaptation was performed in Andeans residing in Bolivia [76]; however to date, there have been fewer studies in Andeans than Tibetans. The peopling of the Andes appears to have begun 12,000 or more years ago [1,2,79], a timeframe that would be expected to permit the natural selection of genes that have at least a modest effect on reproductive success. Just one gene, *EGLN1,* has thus far been identified as being acted upon by natural selection in both Andeans and Tibetans [71]. Among Andeans, several other genes showing evidence of natural selection have been identified, including some involved in vasoregulation (*PRKAA1*, *NOS2*), vascular growth (*VEGFB*, *ELTD1*), cerebral blood flow (*CBS*), and oxidative defense (*FAM213A*) [71,75,77] (Table 2). There has just been one whole-genome sequencing study in Andeans to date; it identified three gene regions—*BRINP3*, *NOS2*, and *TBX5*—with just one (*NOS2*) having been identified previously in a SNP scan [71]. These genes have previously been associated with cardiovascular function, however not hypoxia-sensing [73]. Of note, while we commented above on the role of genetic and developmental factors for the larger lung volumes seen in Andeans, no study to the best of our knowledge has sought to determine the relationship of such morphological variation with any of the gene regions identified as having been acted upon by natural selection.

**Figure 2:**
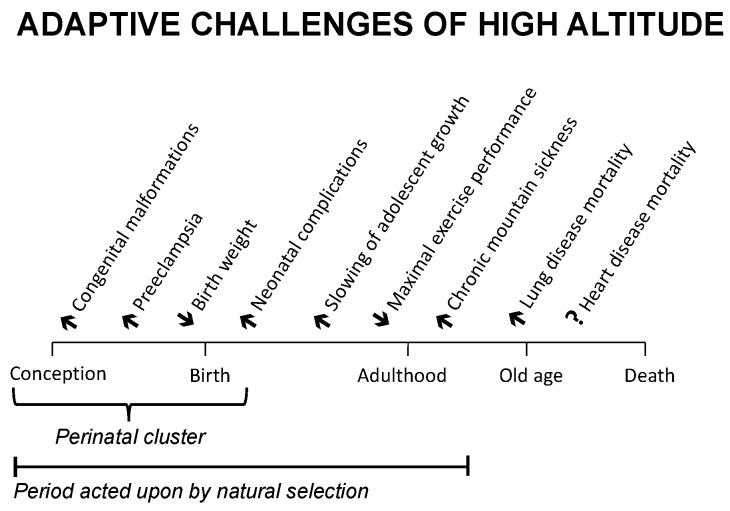
Adaptive challenges or those affecting reproductive success occur at high altitudes at multiple times across the lifespan. About half the cluster during the perinatal period or that from gestation through to the first week of postnatal life, with the remainder occurring during adolescence or adulthood (see text for references). Adapted from [12].

To determine whether genomic regions acted upon by natural selection provide an adaptive advantage in the high-altitude environment, it is essential to understand the functional consequences of the variants identified. Residence at high altitudes poses several challenges for reproductive success; such challenges occur during the perinatal (i.e., from conception through infancy), adolescent, and adult periods, with the heaviest concentration occurring during perinatal life (Figure 2).

#### 2.2.1. Perinatal Complications

**Figure 3:**
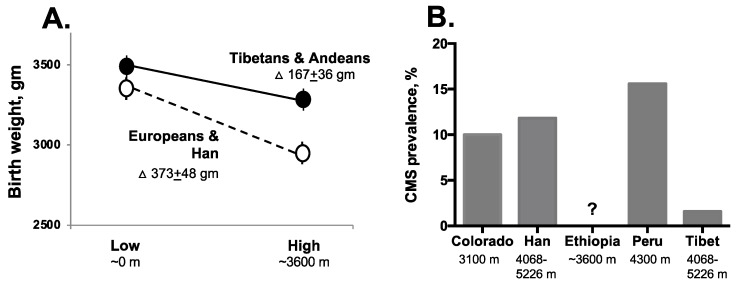
**A.** Tibetans and Andeans have approximately half the altitude-associated reduction in infant birth weight compared with Europeans or Han Chinese (see text for details and [78] for original references). **B**: prevalence of chronic mountain sickness (CMS) is markedly less in Tibetans than similarly aged men from various ancestry groups residing at the altitudes shown. [Adapted from Niermeyer et al. [78] with permission from SAGE Publications.].

Residence at high altitude reduces birth weight as the result of slowed fetal growth rather than shortened gestation [80,81]. Andean infants have half as much birth-weight reduction at high altitude as acclimatized newcomers (Figure 3A), with the magnitude of protection being greater in the Andean populations in the southern (southern Peru, Bolivian) compared to the more northerly region, likely reflecting the duration of high-altitude residence and the extent of forced migration by Incan rulers as well as foreign admixture [82]. Andean protection from altitude-associated reductions in birth weight is directly related to the amount of indigenous American, specifically Andean, ancestry [83,84,85], suggesting genetic involvement. Enhanced uteroplacental blood flow and O_2_ delivery, resulting in part from a larger pregnancy-associated rise in uterine artery diameter [50,51,86,87], is an important factor contributing to Andean protection from fetal growth reductions at high altitude. Numerous studies have shown associations between low uterine artery blood flows, decreased birth weights, and fetal demise [51,88,89,90], supporting the likelihood that maintenance of high uterine artery blood flow is important for normal fetal growth at high altitude. Greater blood flow, not CaO_2_, is responsible for raising uteroplacental O_2_ delivery since CaO_2_ is similar in Andeans and newcomers [51,86]. Placental factors may also be involved since placenta weight, both absolute and relative to fetal weight, is greater in Andeans than acclimatized newcomers [40], and Andean placentas have enhanced villous capillarization and vascular remodeling [91].

High altitude also increases the incidence of preeclampsia, an effect that contributes to the altitude-associated decline in birth weight. Preeclampsia is a multisystem vascular disease of placental origin that complicates roughly 8.5 million pregnancies worldwide each year. It not only poses a threat to maternal and perinatal survival but also increases the risk of cardiovascular disease in affected mothers and offspring later in life [92,93,94,95,96,97]. Existing evidence suggests that high-altitude residence increases the incidence of preeclampsia in Andeans as well as acclimatized newcomers [98,99,100]. However, the lack of systematic assessment of diagnostic criteria and vital-statistic databases in Andean countries has thus far prevented determination as to whether indigenous women are relatively protected compared with acclimatized newcomers. Supporting such a possibility, sFlt-1 levels and sFlt-1/PLGF ratios are lower in pregnant Andeans compared with Europeans living at the same altitudes [53], both of which are protective against preeclampsia [101]. Andeans may also benefit from higher antioxidants levels [52] as well as higher levels of progesterone, estrone, 17-β estradiol, and estriol [55]. Andean pregnant women also have lower cortisol levels than Europeans, with their lower cortisol and higher estriol levels being associated with greater uterine artery diameter and blood flows [55].

Given that compromised fetal growth raises the risk of perinatal mortality, an outcome of direct relevance for reproductive success, we tested the relationship between birth weight and 63 single nucleotide polymorphisms in 16 genes with evidence of natural selection at high altitude while making corrections for multiple comparisons [102]. Several SNPs near *PRKAA1* (coding for the α-1 catalytic subunit of adenosine monophosphate kinase, AMPK) and *EDNRA* (coding the vascular smooth muscle cell endothelin receptor A) were associated with the preservation of birth weight at high altitude; however only *PRKAA1* was also associated with larger uterine artery diameters. In addition, the expression of mTOR-pathway genes in circulating peripheral blood mononuclear cells—a pathway known to play a crucial role in mediating the effects of hypoxia, nutrient restriction, and other factors on fetal growth—differed in women with versus without the selected-for maternal *PRKAA1* genotype, suggesting that AMPK may play an important role in vascular adaptation to pregnancy [103,104,105].

Limited data suggest that native compared with acclimatized newcomer groups have better neonatal outcomes. Perinatal mortality is generally higher at high than low altitudes in South America, with the altitude-associated increase being least in the regions of Peru where populations have lived the longest [82]. Infants of mixed Native American and European ancestry residing at high altitudes in Bolivia spent ~80% of the night with SaO_2_ values below 90%, with lower proportions of the night being seen in children and adolescents [106], but sample sizes and composition were not sufficient to address the possible differences between ancestry groups.

#### 2.2.2. Chronic Mountain Sickness

Slight elevations of red cell mass increase arterial O_2_ content under conditions of ambient hypoxia. In contrast, however, excessive red blood cell production, as observed in chronic mountain sickness (CMS), increases in blood viscosity and impairs blood flow and O_2_ delivery to tissues [73]; for this reason, excessive erythrocytosis is considered to be maladaptive. CMS has long been known to occur at high altitudes [107] and can lead to pulmonary hypertension and right or left heart failure. While such deaths typically occur after the end of the reproductive period, the disease begins in early adulthood and may impact fitness given that affected individuals are no longer able to engage in normal daily activities [108,109].

CMS prevalence varies between highland resident populations (Figure 3B). For instance, CMS has been reported to occur in ~10% of Andean, Coloradan, or Han males over the age of 30 or post-menopausal females, while a smaller proportion of Tibetans are affected [28,110] and, to date, CMS has not yet been reported in Ethiopians [38,111]. CMS has a gradual onset, being seen in 15–25 year old males as preclinical CMS, defined as >2 standard deviations above the mean hemoglobin level or 18.3 g/dL, together with accompanying signs or symptoms [108] and worsening with advancing age [109,112,113]. Persons with CMS have lower levels of ventilation than acclimatized newcomers; however this is also true for healthy Andeans, suggesting that hypoventilation may be necessary yet not sufficient for the development of CMS. Breathing during sleep is likely a key component, with sleep-disordered breathing (apneas, hypopneas) more common in both clinical and preclinical patients [108,109,114,115,116]. Cerebral blood flow is also affected by sleep-disordered breathing [117]. The middle cerebral artery vasodilator response to NO is blunted, carotid artery intimal thickness greater, and flow-mediated brachial artery vasodilation is impaired in Andean men with versus without CMS [118]. Early-life hypoxic exposures may also play a role. Adults with exaggerated hypoxia as neonates showed higher pulmonary arterial pressures during acute-altitude exposure [119]. Compared with healthy controls, high-altitude residents with CMS were more often small-for-gestational age [112], born to a preeclamptic mother, or to have experienced exaggerated neonatal hypoxia [109]. Thus, perhaps hypoxic or oxidative injury during perinatal life predisposes individuals to develop CMS later in life due to impaired pulmonary or cerebral vascular development.

With respect to genetic factors in CMS, *EGLN1*, and *EPAS1*, variants related to hemoglobin levels in Tibetans are not so related in Andeans [120], suggesting that these specific variants may not be involved in increasing Andean susceptibility. A limited whole genome sequencing comparison of 10 men with versus 10 men without CMS identified 11 regions that differed by CMS status [121]. Using a fibroblast cell-culture model, acute hypoxia upregulated two of these genes’ transcriptional responses (*SENP1* and *ANP32D*, known to play roles in regulating erythropoiesis and cellular metabolism, respectively) in CMS patients, but not in controls. The association between *SENP1* and CMS (however not *ANP32D*) was replicated in a larger sample of CMS and control residents of 4338 m [122]. Other genes, such as *SENP1* which codes for a protease that rescues HIF1alpha from degradation, have also been suggested to play a role in increasing susceptibility to CMS [123].

## 3. Speculation on the Role of Epigenetics for Andean High-Altitude Adaptation

Genomic studies are well positioned to reveal functional links between genetic regions that appear to have been subject to recent positive selection and adaptive phenotypes of highland populations. It is critical, however, to recognize that phenotypes are the objects on which selective pressures act and are seldom the product of genetic factors alone. Complex phenotypes most often arise through gene-gene and gene-environment interactions, as well as the functional interaction of the genome and epigenome. Epigenetic marks are non-sequence-based features of the genome that are vital for coordinating transcriptional responses to environmental stimuli. In this way, the epigenome acts as an interface through which genetic sequence is “translated” to generate physiological responses to shifting biological or environmental conditions. This section presents evidence supporting the possibility that epigenetic processes contribute to human high-altitude adaptation, emphasizing the role of epigenetics for transcriptional and developmental responses to limited oxygen availability, epigenetic inheritance, and genome-epigenome interactions. Existing literature largely focuses on transient epigenetic effects. However, several recent investigations have explored mechanisms for epigenetic inheritance and the importance of genome-epigenome interactions for driving physiologic responses and phenotype. Taken together, this work indicates that epigenetic modifications could provide a mechanism for the rapid acquisition of potentially heritable features. This flexibility, itself, could be viewed as a selective advantage during periods of rapid environmental change or periods of the lifespan, such as the perinatal life or pregnancy which require widespread physiological changes over a short time period [124].

Numerous epigenetic mechanisms exist, such as DNA methylation, histone modification, RNA-based mechanisms, and histone variants. DNA methylation, the most well-studied epigenetic modification in humans, is defined by the addition of a methyl group to cytosine residues within CpG dinucleotides. Research has predominantly focused on DNA methylation because of its central involvement in the regulation of gene transcription, genomic imprinting, and the silencing of repetitive DNA elements [125,126]. While the majority of CpG sites across the human genome are methylated [127,128], genomic “islands” of high CpG density (“CpG islands”) are scattered throughout the genome. These regions are generally devoid of methylation, thereby allowing for transcription factor binding and active gene transcription. Hypermethylation of CpG sites within CpG islands typically impedes transcription factor binding, thereby establishing a dormant chromatin state [129]. However, the methylation state of CpG sites within enhancers, gene bodies [130,131], and low-density CpG regions [132] also influences gene expression and alternative splicing.

### 3.1. Epigenetics and Transcriptional Responses to Hypoxia

Epigenetic processes are essential to the regulation of the HIF transcriptional program by, for instance, silencing HIF-stabilization genes, including von Hippel-Lindau (*VHL*) and *EPAS1* [133,134]. De novo methylation of *EPAS1* promoter CpG sites by DNA methyltransferase 3a also prohibits HIF2α-mediated gene expression under hypoxic conditions [134]. DNA methylation events also govern the hypoxic-induction of erythropoietin, a pleiotropic cytokine that is recognized as the central driver of red blood cell production [135]. Moreover, enzymes that alter the epigenetic status of histones and cytosine residues (histone acetyltransferases and demethylases, respectively) are regulated, in part, by hypoxia and are involved in determining chromatin conformation within and around HIF-binding sites [136,137,138]. In this way, epigenomic marks would be expected to influence the “translation” of genomic sequence into physiological responses to acute hypoxic exposure and, potentially, durable phenotypic traits at high altitude [129,139]. Following the same logic, interruption of epigenetic processes that are essential to regulate the HIF-transcriptional program could compromise or augment transcriptional responses that are important to sustain oxygenation under conditions of limited O_2_ supply, such as at high altitude.

### 3.2. Epigenetics and the Developmental Programming of Physiological Responses to Hypoxia

Epigenetic processes are considered central for the effects of intrauterine or early-life exposures on organ system development given the well-established role of epigenetics for determining cellular identity, their responsiveness to environmental and biological cues, and the particular vulnerability of the epigenome to environmental insults in early life. For instance, during embryonic development, the differentiation of genetically-identical pluripotent cells into hundreds of distinct cell types is driven largely via epigenetic mechanisms [140,141]. Existing evidence suggests that the epigenome is involved in the effect of environmental exposures occurring during developmental periods to influence physiological responses to hypoxia in later life. In mice, intrauterine hypoxia induces hypermethylation of CpG motifs located within the protein kinase C epsilon promoter, a gene that encodes a protein known to enhance cardiovascular hemodynamics in ischemia-reperfusion injury, thereby reducing cardiac protein kinase C epsilon expression and, ultimately, increasing the risk of ischemia-reperfusion injury in later life [142,143,144]. Other studies also support the involvement of epigenetic factors for the fetal programming of pulmonary vascular dysfunction. Maternal undernutrition in pregnancy, for example, exaggerates the affected offspring’s pulmonary vascular response to hypoxia and modifies global DNA methylation of the lung [145]. Moreover, treatment of offspring with histone deacetylase inhibitors normalized pulmonary vascular function and DNA methylation status [145].

Existing evidence indicates that perinatal hypoxia may also influence pulmonary vascular function at high altitude in humans. Specifically, lowlanders who experienced transient perinatal hypoxic pulmonary hypertension had an exaggerated pulmonary artery pressure response with high-altitude exposure (4559 m) as adults compared to lowlanders who did not experience hypoxic pulmonary hypertension during perinatal life [119]. Among Andeans residing in La Paz or El Alto, Bolivia (3600–4100 m), adverse oxygenation during perinatal life increases the risk of a preclinical form of CMS and attendant pulmonary vascular dysfunction during young adulthood [109]. Infants born to preeclamptic women at high altitudes also have higher basal pulmonary artery pressure [146]. Further, infants born to preeclamptic women who went on to develop abnormal pulmonary vascular function at high altitude during later life show unique methylation-expression relationships within numerous genes that are important for vascular function, suggesting that epigenetic effects may influence the relationship between pulmonary hypertension and preeclampsia [109]. While much work remains to be done, existing evidence supports the hypothesis that impaired perinatal oxygenation induces epigenetic modifications influencing physiological responses to hypoxia during adulthood.

### 3.3. Inheritance of Epigenetic Marks

From an evolutionary point of view, the relevance of epigenetic marks or the capacity for epigenetic modification for human adaptation depends upon the heritability of epigenetic features themselves and/or the capacity for epigenetic modification in particular regions of the genome. Epigenetic heritability, that is the inheritance of epigenetic marks themselves, remains contentious primarily because non-imprinted genes undergo widespread, yet incomplete, epigenetic reprogramming prior to implantation [147]. However, existing literature supports the persistence of environmentally-induced DNA methylation changes across generations [145,148,149] and the transmission of DNA methylation marks through the germline and somatic pathways [150]. Potential mechanisms for pure transgenerational epigenetic inheritance include constitutional epialleles (epigenetic marks that originate from the early embryo or parental germ line) that are, in some instances, retained across meiotic division [150]. One report further reveals that somatic epigenetic modifications may not need to be carried through the gamete intact, but may rather be transmitted via epigenetic-modifying RNA species [151]. Through this mechanism, heritable DNA methylation marks could avoid the widespread epigenetic reprogramming that occurs during early development. DNA methylation status is also heavily influenced by genetic variation, particularly within CpG motifs [152,153,154,155,156]. One report indicated that up to 80% of genetic variants that disrupt CpG sites alter the methylation status of local CpG sites as well as those located up to 10 kb distant [157].

In short, evidence supporting epigenetic heritability raises novel questions about how genetic sequence orchestrates physiological responses and durable adaptations to environmental exposures such as high altitude. Much work remains to be done in this area, particularly with respect to direct epigenetic inheritance. In the context of human adaptation, understanding the impact of putatively adaptive genetic variants that modify CpG motifs on the epigenetic regulation of gene expression should be of particular interest.

### 3.4. Querying Genomic-Epigenomic Interactions in High-Altitude Populations

As discussed above, SNPs that disrupt (or create) CpG sites are important determinants of epigenetic capacity or, in other words, the potential for epigenetic regulation of gene expression. Prior work has speculated that epigenetics may be involved in high-altitude acclimatization and adaptation [158] and the development of hypoxia-associated pulmonary vascular dysfunction in high-altitude Andeans [159]. However, only three publications report site-specific DNA methylation differences in high-altitude populations [160,161,162], including one paper that presented the hypothesis that genetic variants showing evidence of recent positive selection in high-altitude populations could affect the capacity for the epigenetic modification of gene transcription under hypoxic conditions [162]. Specifically, Julian notes that nearly 40% of the putatively adaptive *EPAS1* SNPs in high-altitude populations modified CpG content [162]. This observation is important in the context of high altitude for several reasons. First, putatively adaptive *EPAS1* SNPs are apparent in high-altitude populations and have been associated with reduced hemoglobin concentrations in some native highland populations (i.e., Tibetans) [163]. Second, the *EPAS1* promoter lies entirely within a CpG island and is epigenetically regulated under hypoxic conditions [134]. Third, *EPAS1* encodes HIF-2α and therefore may be of functional importance for adaptation to hypoxia. Finally, given that CpG-modifying SNPs can influence methylation [157], heritable differences in CpG density may promote or inhibit the epigenetic modifications that influence transcriptional responses to environmental hypoxia. For instance, if a SNP were to decrease CG content in regulatory regions of the genome, there would be less (or no) opportunity for epigenetic regulation of gene expression via DNA methylation (i.e., less plasticity). Alternatively, a SNP that increased CG content in regulatory regions may be more permissive of epigenetic regulation via DNA methylation (i.e., more plasticity). While this concept is provocative, it is also somewhat premature and requires further investigation.

Epigenomic processes may also contribute to maladaptive phenotypes of high-altitude populations, including CMS in highland Andeans. Julian contrasted peripheral blood mononuclear cell DNA methylation patterns between Andean men living in La Paz-El Alto, Bolivia who presented with a preclinical form of CMS and healthy controls [162]. Of the numerous differentially methylated regions identified, the most notable differentially methylated region (DMR) associated with preclinical CMS was the hypermethylation of *EGLN1* [162], a gene that encodes PHD2. Given that PHD2 negatively regulates the HIF-transcriptional program via promoting the proteasomal degradation of HIF1/2α [67], the hypermethylation of *EGLN1* would be anticipated to diminish PHD2 expression and thereby enable the transcription of HIF-regulated genes such as erythropoietin. In support of the hypothesis that hypermethylation of *EGLN1* contributes to the excessive production of red blood cells in CMS, *EGLN1* inactivation in mice results in an overproduction of erythropoietin and polycythemia, and familial polycythemia in humans has been linked to *EGLN1* mutations [164,165,166]. Further investigations are needed to not only test this hypothesis, however also to evaluate the functional importance of the DMRs identified.

## 4. Summary, Conclusions, and Directions for Future Work

In summary, there are several unique O_2_-transport characteristics of Andeans compared to acclimatized or lifelong newcomer residents of high altitude. As reviewed above, these are lower alveolar ventilation, lower hypoxic pulmonary vasoconstrictor response, slightly larger lung volumes, higher uterine artery and possibly lower middle cerebral blood flows, less altitude decrement in maximal exercise O_2_ consumption, and more efficient cardiac O_2_ utilization. Collectively, these are suggestive of greater efficiency in O_2_ transfer and utilization; in turn, such differences between acclimatized or lifelong high-altitude residents support the existence of Andean genetic adaptation to high altitude.

Direct support for Andean genetic adaptation to high altitude comes from SNP genome scans and whole-genome sequencing studies. Genome scans can be performed at relatively low cost and in large numbers of persons, but only sample a small portion of the genome [1]. They have shown that natural selection has acted on a gene region that is involved in regulating the HIF-pathway, *EGLN1*, and on others that are not in the HIF-pathway yet are O_2_ sensitive, underscoring the importance of looking broadly at the range of genetic factors potentially involved. Whereas whole-genome scans are necessarily more complete, they are considerably more expensive and hence difficult to conduct in large numbers of persons. Thus more, especially high-coverage whole-genome sequencing studies, are needed. The one whole-genome scan to date indicates, intriguingly, that selection has acted not only on genes that are involved in O_2_ sensing, but also on those regulating cardiovascular responses to hypoxia [73].

Future studies are also required to provide deeper exploration of the associations between selected-for genotypes and phenotypic traits that are likely to influence reproductive success. The inclusion of epigenomic factors in such studies is also vital as the few studies conducted to date indicate potentially key roles for epigenetic regulation of gene transcription in ways that could affect reproductive fitness. While functional studies on the impact of locus-specific methylation status remains challenging, the advent, for example, of genome-editing technologies such as the CRISPR/Cas-9 system permit the induction of targeted CpG methylation and demethylation events in vitro as well as in vivo experimental animal models [167]. Transcription Activator-Like Effector Nucleases (TALENs), another genome-editing technique, can also be used to target locus-specific CpG methylation sites [168,169]. Using these strategies, future experimental models could be developed to determine whether hypomethylation or hypermethylation of specific CpG sites affect molecular and, ultimately, physiological function.

In short, the singular nature of the hypoxic stress posed by residence at high altitudes together with the central role played by oxygenation for health and disease states during intrauterine and postnatal life continues to provide a unique study environment for advancing our understanding of the mechanisms underlying human adaptive potential and of human evolutionary processes. Ultimately, such studies can also benefit biomedical research with the identification of new therapeutic targets for treating or preventing O_2_ related diseases.

## Figures and Tables

**Table 1:** Determinants of O_2_ transport in long-term highland groups and acclimatized newcomers at ~3600–4300 m.

Variable	Acclimatized Newcomer	Andean	Andean versus Accl newcomer
P_A_CO_2_, mmHg	30 [8]	Higher [8,32]	↑ω
A-a O_2_, mmHg	7–11 [14,15]	Lower [4,15]	↓
SaO_2_, %	92 [16,19,37]	Same [9,37,38,39,40]	≅
Hemoglobin, g/dL	17.6 [16,19]	Same [9,17,21]	≅
CaO_2_, vol%^11^	21 [16,19]	Same [19]	≅
Ppa hypoxic response	Present	Intermediate [41]	↓
Brain blood flow velocity, cm/s	27 [42]	18% [39]	↓
Uterine artery blood flow, mL/min	269 [43]	Higher [43]	↑

Abbreviations: A-a DO_2_ = alveolar to arterial O_2_ diffusion gradient, Accl = acclimatized, CaO_2_ = arterial O_2_ content, P_A_CO_2_ or P_ET_CO_2_ = alveolar or end-tidal PCO_2_, Ppa = pulmonary arterial pressure, SaO_2_ = arterial O_2_ saturation. Numbers in the table are mean values or, in cases where few data are available, ranges. References are given in parentheses.

**Table 2:** Autosomal gene regions acted upon by natural selection in Andean populations.

*AS3MT* [72] *BRINP3* [73] *CLC* [72] *DUOX2* [72]	*EDNRA* [71,74] *EGLN1* [71,74] *ELTD1* [75] *ET-1* [76] *FAM213A* [77]	*NOS2* [71,73,74] *PRKAA1* [71,74] *SFTPD* [77] *SP100* [72]	*TBX5* [73] *TMEM38B* [72] TP53 pathway [78] *VEGFB* [75]

Abbreviations: *AS3MT* = arsenite 3 methyltransferase; *BRINP3* = BMP/retinoic acid inducible neural specific 3; *CLC* = galectin-10; *DUOX2* = dual oxidase 2; *EDNRA* = endothelin receptor type A; *EGLN1* = egl-9 family hypoxia inducible factor 1; *ELTD1* = adhesion G protein-coupled receptor L4; FAM213A = family with sequence similarity 213 member A; *NOS2* = nitric oxide synthase 2; *PRKAA1* = protein kinase AMP-activated, α 1 catalytic subunit; *SFTPD* = surfactant protein D; *SP100* = SP100 nuclear antigen; *TBX5* = T-box 5; *TMEM38B* = transmembrane protein 38B; *TP53* = tumor protein p53; *VEGFB* = vascular endothelial growth factor B.
